# Task allocation in multi-robot system using resource sharing with dynamic threshold approach

**DOI:** 10.1371/journal.pone.0267982

**Published:** 2022-05-04

**Authors:** Nayyer Fazal, Muhammad Tahir Khan, Shahzad Anwar, Javaid Iqbal, Shahbaz Khan

**Affiliations:** 1 Department of Mechatronics Engineering, UET Peshawar, Peshawar, Pakistan; 2 College of Electrical and Mechanical Engineering, NUST, Islamabad, Pakistan; 3 Institute of Manufacturing Engineering Management, University of Engineering and Applied Sciences, Swat, Pakistan; Universiti Sains Malaysia, MALAYSIA

## Abstract

Task allocation is a fundamental requirement for multi-robot systems working in dynamic environments. An efficient task allocation algorithm allows the robots to adjust their behavior in response to environmental changes such as fault occurrences, or other robots’ actions to increase overall system performance. To address these challenges, this paper presents a Task Allocation technique based on a threshold level which is an accumulative value aggregated by a centralized unit using the Task-Robot ratio and the number of the available resource in the system. The threshold level serves as a reference for task acceptance and the task acceptance occurs despite resource shortage. The deficient resources for the accepted task are acquired through an auction process using objective minimization. Despite resource shortage, task acceptance occurs. The threshold approach and the objective minimization in the auction process reduce the overall completion time and increase the system’s resource utilization up to 96%, which is demonstrated theoretically and validated through simulations and real experimentation.

## 1.Introduction

Task Allocations are considered in a range of applications, such as path exploration [1], path planning [2], foraging [3], tracking [4], and transportation [5], and have also been applied to many domains like industrial engineering [6]. With the increase in demand for multi-robot cooperation in complex and uncertain environments, the significance of the Task Allocation in Multi-Robot systems, i.e., to determine an efficient task assignment to improve the system efficiency [7] and task completion time [8–11] has increased. A challenge in using teams of robots is the cooperation of robots to perform tasks while optimizing one or more objectives. Each application has different requirements for Task allocation, as in search and rescue operations, the survivors need to be assisted within specified deadlines, where the objectives are to maximize the number of survivors and minimize the average waiting time of their rescue. Similarly, tasks in the manufacturing process require task completion with different constraints. The optimization objectives usually include completing all the tasks in the shortest possible time. As opposed to the manufacturing environment, the search and rescue operations normally deal with the unstructured environment, highly dynamic conditions, and limited resources. Hence the Task Allocation techniques and objective function requirements may vary with application and different frameworks. For example, a Centralized system offers a clear chain of command, a focused vision, and quick implementation using the global information and optimization-based heuristics to generate global solutions for task allocation [12–14]. However, it lacks the agility about robustness, scalability, and flexibility in dynamic circumstances.

**Fig 1:**
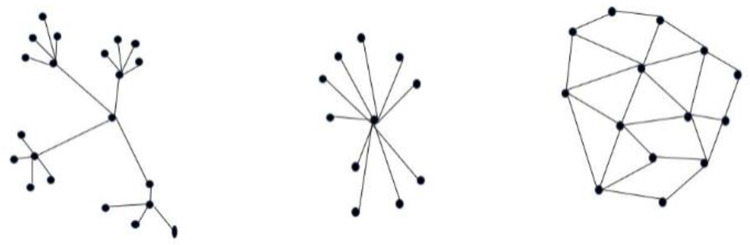
(a). Decentralized (b) Centralized (c) Distributed.

On the other hand, distributed control is generally complex to implement and lacks global knowledge of the system’s current status. Hence to attain the benefits of different frameworks, this study aim to develop a decentralized system. In a decentralized approach there is no single point where the decision is made. Instead, each node decides its own behaviour and the overall behavior is an aggregate response of all the node’s response, as shown in Fig 1 for demonstration.

To develop a decentralized system a dynamic Adaptive Inception Value (*AIV*(*i*)) is introduced here, which is generated by accumulating the entire system status by aggregating the number of tasks, number of robots, and the faults occurrence or resource absence in the system. The robot uses this aggregated value as a reference for task acceptance. When a robot encounters a task and the resource deficiency relevant to the detected task is not more than the acceptable deficiency level that is determined through *AIV*(*i*), the robot becomes an auctioneer to acquire the deficient resources. The task allocation and completion times are further improved by incorporating objective minimization in the bidding process. The auctioneer can settle its main concern of either time minimization or thedistance travelled by the robot by minimizing the corresponding objective value.

Extensive work has been performed in the field of task allocation in multi-robot systems; however the focus is always on the bid calculation, and the winner selected by the auctioneer, also the tasks are supposed to be assigned by a centralized auctioneer to the robot that matches the task criterion which increases the computation time of task allocation. Moreover, the auctioneer selection, the resource absence, or resource failure and its effect on auctioneer selection has never been addressed. This paper aims to develop a methodology for task allocation which can easily adapt the changes with less computation time for task allocation and allows any robot in the system to become an auctioneer if its deficiency is below the threshold level as settled by the central monitoring. The proposed approach reduces the computation time of task allocation and, consequently, task completion time and the system’s communication burden.

The remaining of the paper is organized as follows, different techniques used for task allocation and the gap analysis are presented in Section 2. To overcome the gap, the proposed methodology is proposed in Section 3. To validate the performance of the proposed method, simulation and real experimentation have been performed in Section 4, whereas the results obtained and validated with benchmark techniques are given in Section 5. Finally, the conclusion of the proposed method and results and the future work are given in Section 6.

## 2.Related work

The literature on task allocation for multi-robot systems(MRS) is very broad [15,16]. Task allocation mechanisms aim to allocate tasks to the appropriate robot in the most optimal way based on one or more objectives. In [15], a complete organization of task allocation problems is delivered, which is repeatedly used in the task allocation literature. Usually, two approaches are employed to solve task allocation problems in literature: market-based approaches and optimization-based approaches [17,18]. The existing work on each of these two approaches is explained as follows:

### Optimization approach

The optimization-based approach is usually employed in a centralized system where one centralized point can access the global information and find the optimal global solution for task allocation. This type of NP-hard optimization problem is generally solved using metaheuristics or heuristics. The optimization algorithms that are frequently in use for task allocation are Ant Colony Optimization [19], Simulated Annealing [20], and Genetic Algorithms [21].

A broader review is presented in [22] on different techniques and algorithms of optimization. A team of heterogeneous or homogeneous robots can cover more area and could be more robust to failures than a single all-purpose robot [23,24]. The dynamics of collaboration between the robot and the workers in a shared workplace and its impact on task allocation are key parameters for optimal solutions [25]. The collaboration among many robots has been employed in [26] to make a coalition of all the targets for the sequence in which UAVs need to be assigned to the task to get a minimal time of completion. Sometimes a single optimal allocation plan may not work efficiently as the uncertain emergencies may frequently occur during real execution processes of multi-robot cooperation, and the predefined optimal task allocation techniques may not work as expected. To tackle different forms of failures, outstanding work has been performed. For the disputation of resources, the interference issue is addressed when multiple robots content for the same resource [27]. An ontology-based mechanism is proposed in [28] for the fluctuating coupling affairs. Still, these techniques can only handle specific types of measures. An optimal assignment strategy is desired, such that the scheme can switch to another solution when it cannot continue anymore. Usually, more robots are available for limited tasks, but the problem arises with robots and resource constraints and the tasks are time-bound. The multiple optimal solutions are normally employed in manufacturing and have highly valued practical applications. Second, the uncertain failures in the system and the physical and energy constraints impact the long-term execution. Also, multiple solutions with similar or the same quality are important for the flexibility and robustness of a system. Hence, as premeditated by a centralized unit, the multiple optimal solutions can act as the prior information for a distributed multi-robot system to improve the cooperative work and reduce the chances of deadlock and resource conflict.

The Task allocation based on optimization techniques works well for simple and small systems but usually lacks optimality for larger and complex systems. The number of tasks and robots increase the computational complexity exponentially. The user usually limits the number of iterations for an optimization solver to bound the execution time of the process to compute more task allocations in the limited time, which allows a faster response to dynamic changes. By limiting the optimization time, scarcer solutions are explored by the algorithm. This prevents the algorithm from fully exploring the search space, resulting in depraved optimization and giving sub-optimal solutions to more complex and larger systems. The decentralized market-based task allocation approaches could be used as an alternative that is used in this paper as a solution.

### Market based approach

The computational time of the Market-based approach may scale better with the problem complexity and overcome the limitations of optimization-based approaches. The competence of auction-based algorithms has been proved in [29,30]. A market-based approach is a decentralized optimization that uses an auction method with bids to attain an assignment. Generally, an auctioneer announces the task to all the robots, and in response, the robots compute bids on tasks and send it back, and the auctioneer allocates the task to the robot with the lowest bid. This very basic auction-based protocol is called the CNET protocol [31]. Other variations to this protocol are deeply investigated, such as parallel single-item auctions, where every robot computes a bid for each task. The auctioneer assigns all tasks at once. The solutions are ecpected to be highly sub-optimal since it does not account any interaction between tasks [32]. Another method is the combinatorial auctions where every robot computes a bid for each subset of the task on offer. By considering the possible combination of tasks in the bidding process, an interaction is established [32]. The combinatorial auction method usually produces solutions that are close to optimal but need a very high computation time. A compromise between the parallel and combinatorial auctions is a Sequential single-item auction for computational effort and optimality. The auction continues for several rounds, and in each round, a task is assigned to a robot. The Sequential single-item auctions, in general, do not guarantee to find an optimal solution. However, synergies are exploited between tasks.

The most challenging problem in multi-robot systems is to optimally assign robots to tasks that optimize the overall system performance and employ more than one robot for a task. Multi-robot task allocation is a complex problem for heterogeneous robots with different capabilities and unreliable resources that are to perform tasks with different constraints and requirements in an optimal way and to attain the benefits of both centralized and distributed frameworks. Hence, the authors here use the market-based approach to select an optimal solution proposed by the bidder robots that minimizes the given objectives. The proposed approach also selects the auctioneers in the system using the system status as a criterion which may generate multiple parallel auctions in the system.

## 3. Methodology

In our proposed approach for task allocation using resource sharing, each robot has to serve transportation services. The overall approach is termed a decentralized approach where each robot is independent to decide, but the resulting system behaviour is the accumulation of all robot’s response as shown in Fig 1(A). An accumulative value is generated by the centralized unit used as reference value for task acceptance by the robots. Although it is arguable to refer to the proposed system as centralized or decentralized, the authors of this paper termed it a decentralized system.

The tasks are accepted by the robots based on reference/ threshold value (*AIV*(*i*)) set by the centralized unit whereas the deficient resources are acquired from other robots using Market-based approach.

The different auction principles for the Market-based approach are,

- In a Parallel single-item auction, each robot computes its bid for all the tasks, and the auctioneer allocates all the tasks at once
- In a combinatorial auction,each robot computes a bid for every subset of tasks on-demand.of tasks on demand in the combinatorial auction. By considering the combination between the tasks in bidding process a combined effect is produced.
- The sequential single-item auction is a compromise between combinatorial and parallel auctions in computational effort and optimality. One resource sharing request is granted in each round. Literature [32–34] establishes that the sequential single-item auction gives best trade-off between simplicity and optimality.

Whenever there is a shortage of resources relative to the encountered task and the deficiency of the robot is within the frontiers of *AIV*(*i*), the robot becomes an auctioneer and announces the resource shortage to the system’s robots. Then, the bidder robots send the Sealed first-price auctions [35], where all robots place their bids secretly without the knowledge of other robot’s bids, and the auctioneer selects the best bid.

### 3.1 Task robot encounter

**Fig 2:**
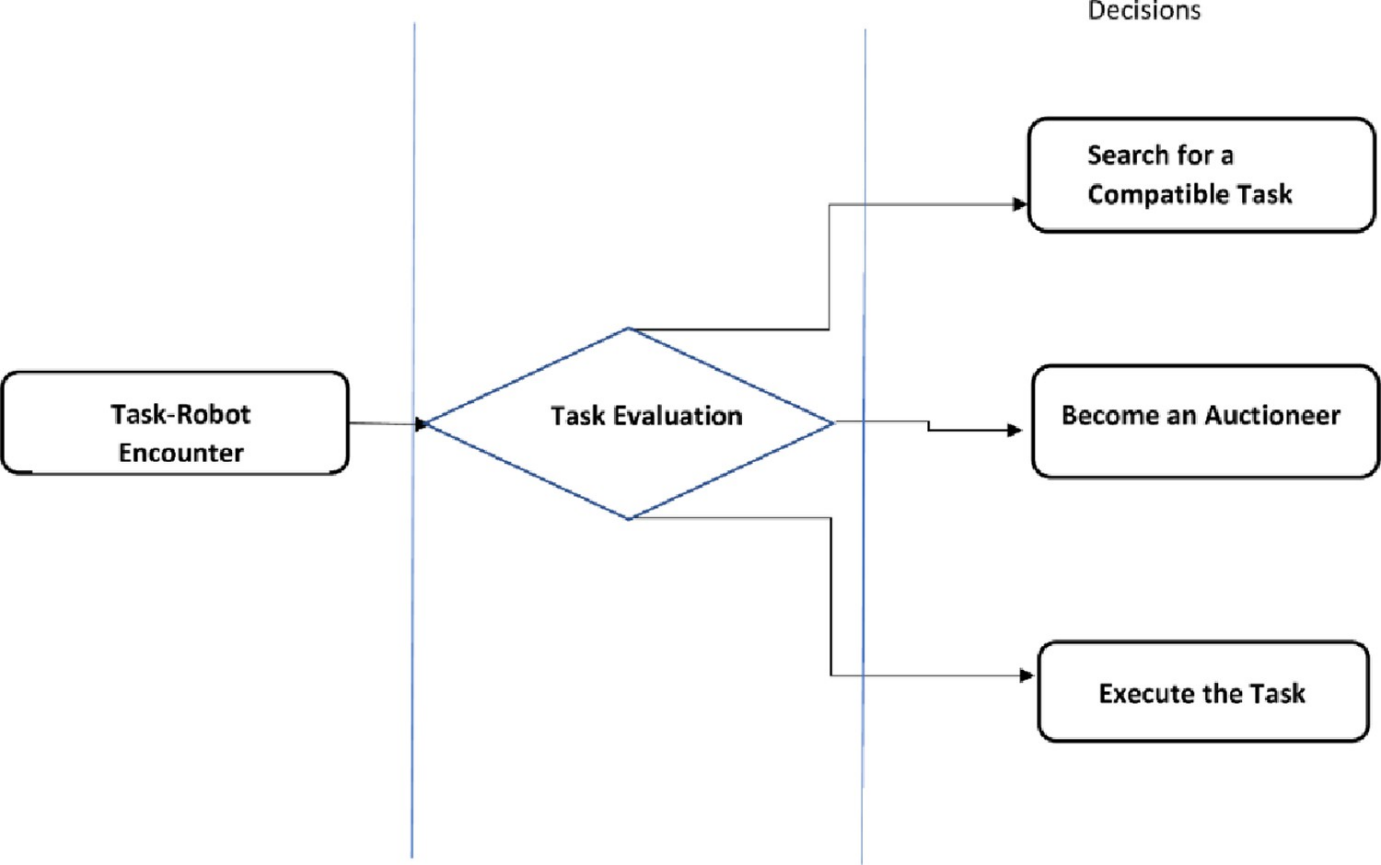
Generalized methodology for the proposed resource sharing mechanism.

The system starts with a specific number of resources, the number of tasks, and the number of robots. Then, the robots randomly search for a task. When the robot has detected a task, it evaluates the task with its available resources and compares its deficiency with the acceptable deficiency level settled by the Centralized unit,as depicted in Fig 2. The tasks are considered as purely transportation tasks where each robot should detect and recognize the task and place it at the designated location. Table 1 gives a list of abbreviations used in the methodology.

**Table 1:** List of abbreviations used in the methodology.

Abbreviation	Description
*AIV*(*i*)	*Threshold level set by the centralized unit at instant i*
*r*_*n*_	*Resources of robot*
ȓ	*Resource requirment of the task*
*T*_*k*_	*k*^*th*^ *task*
*F*_*nk*_	*Fitness of robot n for task k*
*ς*	*scarcity of robtot n for task k*
*Deff*_*k*_	*Acceptable defficiency level for task k*

**Fig 3:**
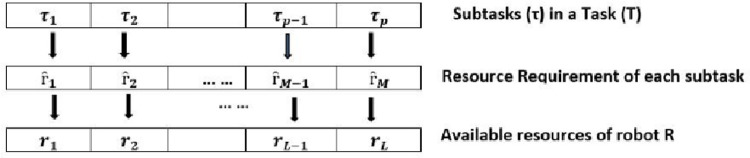
Mapping of subtask’s requirement with available resources of the robot.

Fig 3 shows the mapping of resource requirement of each task to the resources of a robot which identifies the unavailable resources of the robot and may demand other robots in the system provided it satisfies the criteria, described in the forthcoming section.

The resources of the *n*^*th*^ robot is represented with *r*_*n*_, given as

$$r_{n}=\{r_{1},r_{2},\ldots r_{L}\}$$ (1)

*r*_*L*_ denotes the maximum number of working/operational resources of the robot.

The Resource Requirement matrix of the task is represented as given in Eq (2)

$$ȓ=\{{ȓ}_{1},{ȓ}_{2}\ldots {ȓ}_{M}\}$$ (2)

Each subtask has a different resource requirement. When a robot detects a task, it maps its available resources with the resource requirement of the task, as shown in Fig 3. Where each task has several Subtasks, represented in Eq (3)

$${T=\{\tau }_{1},\tau_{2}\ldots \tau_{P}\}$$ (3)

The fitness of the robot to perform the subtask of the detected task is 1 if the resource relevant to the subtask is available and “0” otherwise, represented in Eq (4)

$$ʄ(R_{n},\tau_{p_{T_{k}}})=\left\{\begin{array}{l} 1\;if\;{r_{\tau_{p}}}_{T_{k}}є\;r_{k}\\ \;0\;Otherwise \end{array}\right\}$$ (4)

where $\tau_{p_{T_{k}}}$ represents the *p*^*th*^ subtask of task *k*, and ${r_{\tau_{p}}}_{T_{k}}$ represents the resource required for *p*^*th*^ subtask of the *k*^*th*^ task

The accumulative fitness of *n*^*th*^ robot for the *k*^*th*^ task is computed using Eq (5)

$$F_{nk}=\sum_{p=1}^{P}ʄ(R_{n},\tau_{p_{T_{k}}})$$ (5)

**Fig 4:**
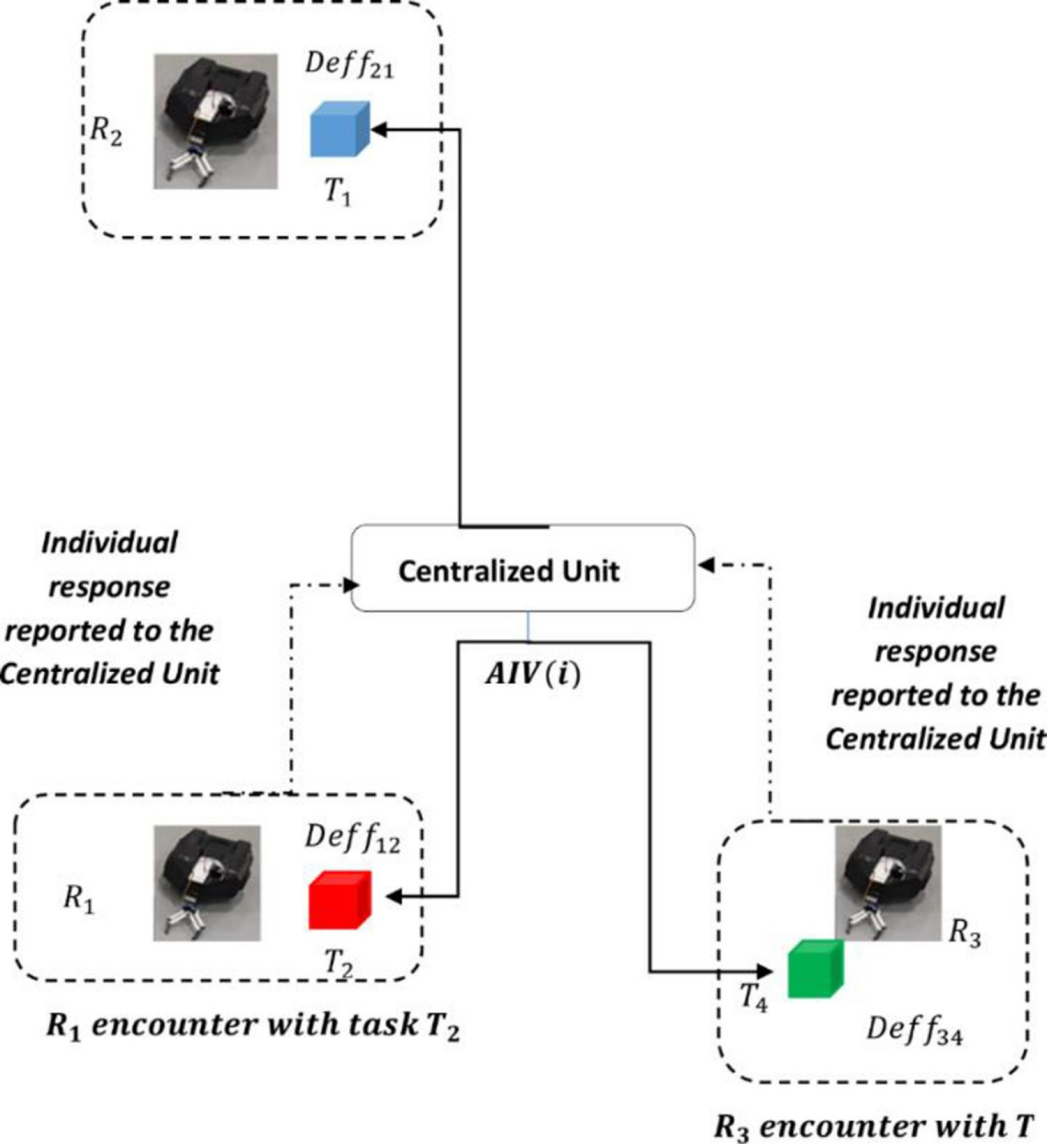
Generalized decentralized task-robot encounter and system status aggregation depiction.

For the same AI*V*(*i*), each Task-Robot encounter has a different fitness value. The robot computes the fitness value for each task-robot encounter and each encounter accumulates and gives an aggregate status of the system. Once the fitness is evaluated, the robot computes its scarcity level relative to the task, which is the difference between the fitness value and resource requirement. A generalized task-robot encounter is depicted in Fig 4.


$$\varsigma =|F_{nk}-ȓ|$$ (6)

The *ς* (scarcity) categorizes the subsequent stage of the task, explained in the forthcoming, Task Evaluation section.

### 3.2 Task evaluation

For task evaluation, the robot compares its scarcity level with the *AIV*(*i*), which serves as a threshold level for the instant *i*, defined by the central unit.

The Central Unit computes the *AIV*(*i*) by considering the Task-Robot ratio and the number of operational resources in the system. The initial Task-Robot proportion is given as Eq (7)

TRi=TinRin (7)

Where the average resources per robot are computed as in Eq (8), the *r*_*in*_ represents the initial total resources in the system

Ꝣ=rinRin (8)

When there is a change in the total number of resources that might occur due to resource failure, the average resources per robot, named Operational robots, changes. The Operational Robots are calculated using Eq (9)

Ropr=roprꝢ (9)

This *R*_*opr*_ helps determine the total effective operational robots in the system and revises the Task-Robot ratio.

The instantaneous value of the task has also been modified in Eq (10), as some of the tasks might have ended with completion, which results in changing the total number of tasks at the instant *i* as *T*_*i*_. For *Normalization*,

TRiTRmax=TiRoprTRmax (10)

Hence using Eq (10), the central monitor updates the Adaptive Incentive Value (AIV),

$$AIV(i)=\frac{T_{i}.Ꝣ}{{TR}_{max}.}$$ (11)

The *AIV*(*i*) accumulates the entire system status into a single dynamic parameter updated with each fault occurrence or task completion in the system. The *AIV*(*i*) is used as a reference value for task acceptance. The robot is allowed to accept the task, despite resource shortage and, can become an auctioneer to acquire the deficient resources through resource sharing when the deficiency level does not exceed the reference *AIV*(*i*). Adopting this approach ensures realistic resource sharing with reasonable resource switching among the robots, as excessive switching may also degrade system performance. The *AIV*(*i*) computed by the centralized unit is globally known to all the robots and computes the acceptable deficiency level of the robot for the encountered task as given in Eq (12)

$${Deff}_{k}={ȓ}_{k}.AIV(i)$$ (12)

Eq (12) gives the acceptable deficiency level for a task. The decision making is explained using Eqs (6) and (12).

The AIV affects the system in the following way,

With a higher Task-Robot ratio, the task acceptance rate is high, which increases resource sharing in the system in order to increase the overall number of task completion and a greater number of robots become auctioneers.

Whereas when Task-Robot ratio is lower and with fewer faults reported in the system, the robot focuses on improving the quality of performance of the task by accepting tasks with a higher fitness value hence engaging fewer numbers of robots in performing a task.

As an explanatory example, at a value of *AIV*(*i*) = 0.2 and a task requirement ȓ_*k*_ = 10, the robots can accept the task if 80% of the resources relevant to the detected task and acquire the remaining 20% using resource sharing through the auction process. The actual deficiency of the robot relevant to the detected task is calculated using Eq (6).

### 3.3 Decision making

- When (*ς*≥*Deff*_*k*_), the robot ignores the task as the robot’s deficiency to the relevant task is less than the acceptable deficiency level and the robot searches for another task.
- When the robot’s resources match the resource requirement of the task and (*ς* = 0), Eq (6), the robot performs the detected task
- When *ς*≤*Deff*_*k*_, the robot’s deficiency to the detected task is less than or equal to the acceptable deficiency level of the task, the robot completes the detected task by becoming an auctioneer and requesting other robots in the system to share their surplus resources.

### 3.4 Auction process

#### 3.4.1 Bidder’s role

To participate in the auctions, the robot needs to know its location, its local task list, its resource engagement, and the resource requirement of the newly detected task. The robot computes the cost in terms of time and sends their bids using two of the rules available in [36], the MiniSum and MiniMax. The MiniSum computes the extra cost for utilizing its resources with the newly announced task in its task list where the task is inserted in the task list using the optimal insertion heuristic [37]. For this purpose, the robot first computes the cost for the resource usage, for the tasks that are already assigned as *C*_1_ and the cost with the newly announced task as *C*_2_ and figures out its difference (*β*_*ms*_ = *C*_2_−*C*_1_)

Whereas MiniMax computes the total cost for the already assigned task and the newly announced task with no marginal cost. Consequently, the MiniSum tends to minimize the total path cost of overall robots, whereas the MiniMax reduces the total time of tasks execution. Both approaches have their paybacks in certain situations. Once these bids are calculated the total bid is sent to the auctioneer using a linear combination of both as given in the following Equation

$$\beta_{tot}=(1-\alpha )\beta_{mm}+\alpha \beta_{ms}$$ (13)

To prioritize the effect of one of these as per requirement, another tunable parameter α is introduced here. When the Task-Robot ratio is higher than a specific ratio and the tasks need to be completed within a specific time, then α = 0, whereas when there are fewer tasks in the system, and the goal is to reduce the total cost, then α = 1.When both the objectives are minimized simultaneously the value of α is set at 0.5.

#### 3.4.2. Auctioneer’s role

The rule for the bid selection by the auctioneer can alter [16]. The simple Lowest Bid has been used here where the auctioneer computes the bids sent by all the bidders and accept the resource offered by the robot which has sent the lowest bid. A combination of our auctioneer selection rule and the Lowest Bid principle minimizes the total cost and the total time in which all the tasks are executed by selecting optimal solutions.

### 3.5 Task-robot architecture

We have two entities in Resource Sharing based task allocation: the auctioneer and the bidder. Here we introduce two task-robot architectures, one which assumes the bidding rule algorithm, and the other which determines the winner.

#### 3.5.1 Bidder algorithm

**Fig 5:**
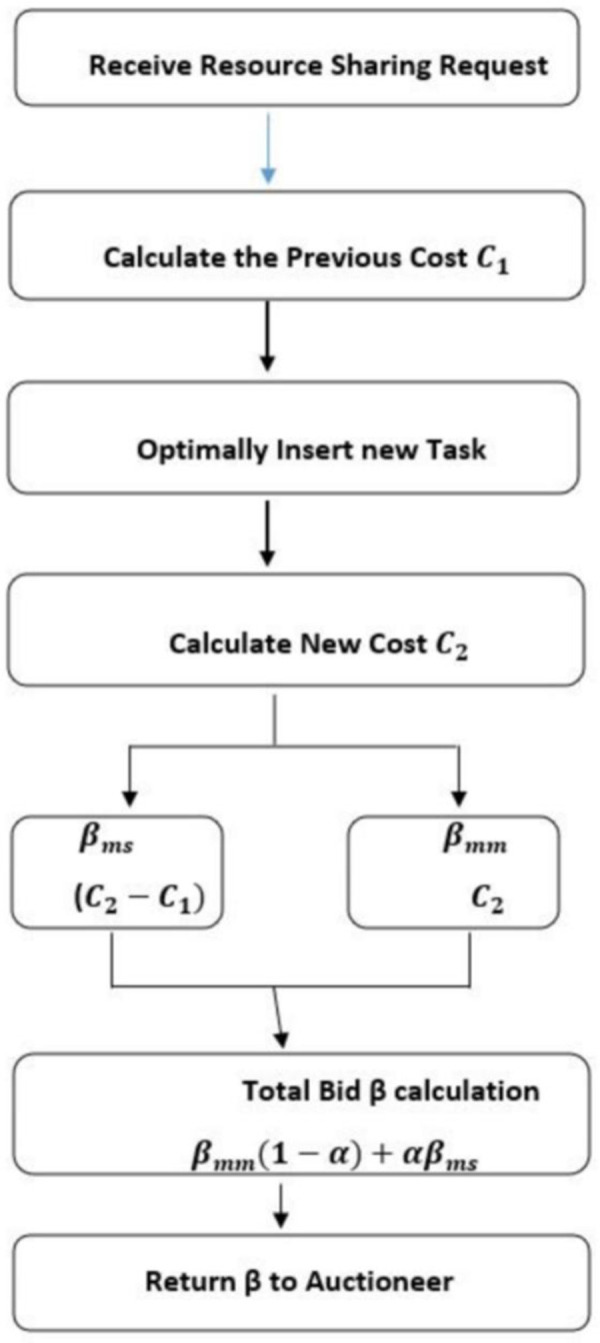
Bid calculation.

The region of the announcement is generally considered for large-scale systems. In the proposed approach, when a robot becomes an auctioneer, the auctioneer announces the task to all robots in the system as shown in Fig 5. In the case of very large scale systems, this may require many unnecessary computations as those robots

That is very far from the auctioneer and needs to compute the bids. However, in the case of sharing the processing capabilities of the robot, the distance between the robots seldom matters.

#### 3.5.2 Auctioneer’s algorithm

**Fig 6:**
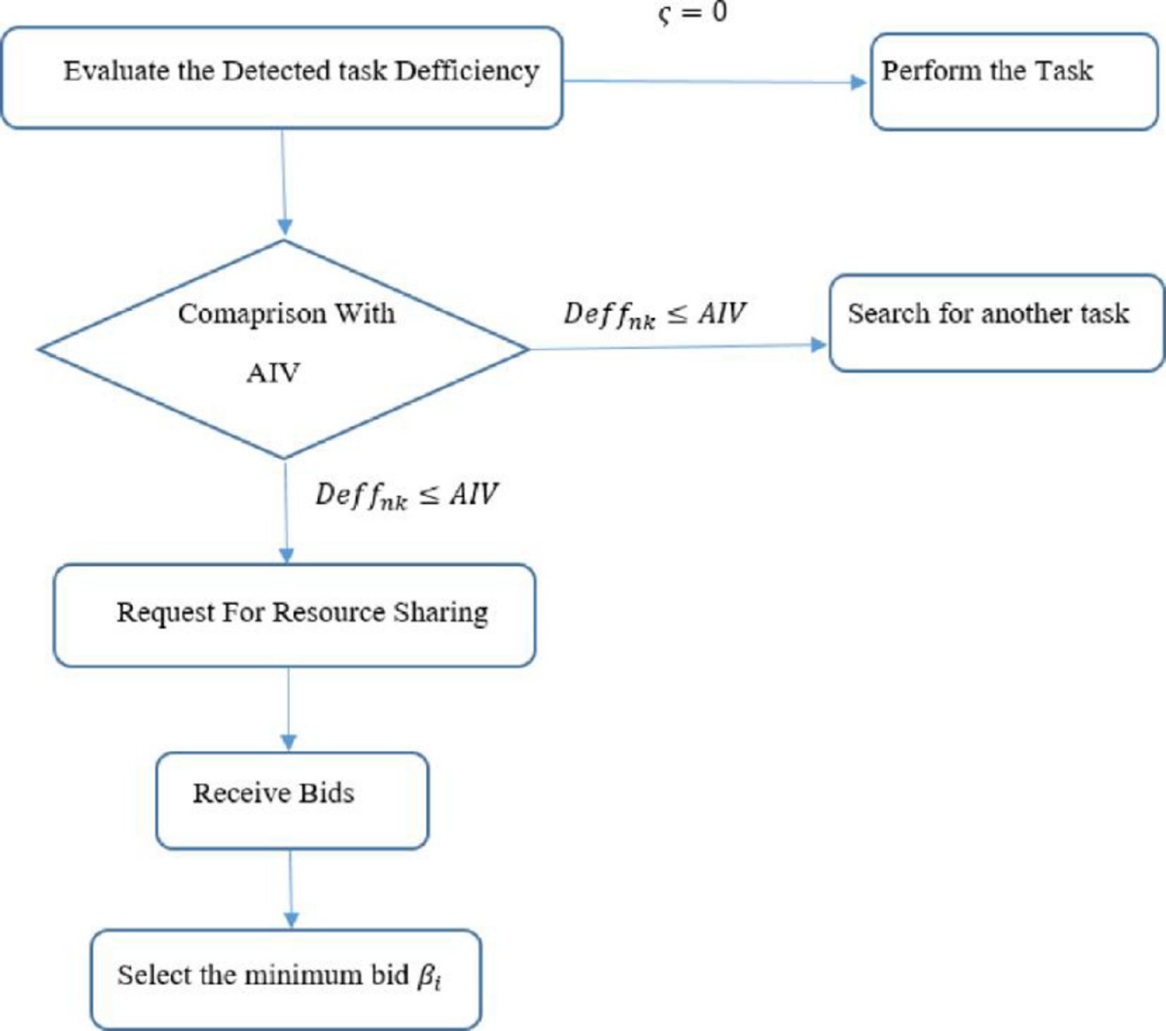
Auctioneer’s role.

The role of the auctioneer can be assigned on be token-based or time-based mechanism. The authors here have used the *AIV*(*i*) as a reference for the dynamic criterion for auctioneer selection which is announced by the centralized unit and changes in the system status. If the robot’s deficiency, relevant to the detected task is less than or equal to the acceptable deficiency level *Deff*_*nk*_, computed through *AIV*(*i*), the robot becomes an auctioneer and requests deficient resources depicted in Fig 6.

## 4.Experimentation

To validate the proposed mechanism, a manufacturing warehouse was formulated with heterogeneous multi robots that were supposed to detect and recognize the task and transport it to the goal location. Tasks of different colours (red, green, blue) and different shapes as shown in Fig 5 were introduced for the robots to classify and preserve them at different warehouse locations. Each robot was supposed to have capabilities for task detection and transportation, such as RGB sensor, Camera, image recognition, task organization to the goal location, the Gripper, and the Bumper for pushing the task. Furthermore, random resource absence and faults were introduced to see their effect on task allocation and performance. Resource sharing was classified into two categories.

- One which needed physical movement of the robot to share its resources such as RGB sensor, Camera, Gripper and Bumper
- The other which did not need any physical movement for sharing resources such as image recognition and task organization, where resources could be shared remotely

Each robot had a certain battery level and local task list that contained the tasks assigned to the robots. The Task-Robot ratio was also varied for different scenarios.

### Parameters used

The parameters listed in Table 2 are selected for both the simulations and real experimentation. The same parameters were considered for comparison with baseline techniques.

**Table 2:** Parameters used for experimentation.

Parameter	Value
Simulation time step	0.002s
Camera frame rate	10Hz
Camera resolution	640*640
Number of tasks	3–36
Area	6m*6m
Goal location	3
Velocity of robot	0.20m/s
Robots in real experimentation	3
Robots in simulation	4

The number of task were varied from 3–36,with number of robots 4 for simulation and 3 for real experimentation to validate the fact that the performance depends on the task-robot ratio and not only the total robots in the system. The performance remained the same when the tasks were increased beyond 36The camera frame rate was set at 10HZ in both the real and simulation setup because a very high frame rate could increase the image processing load and could introduce additional delays in the process.

### 4.1 Simulation experiment

**Fig 7:**
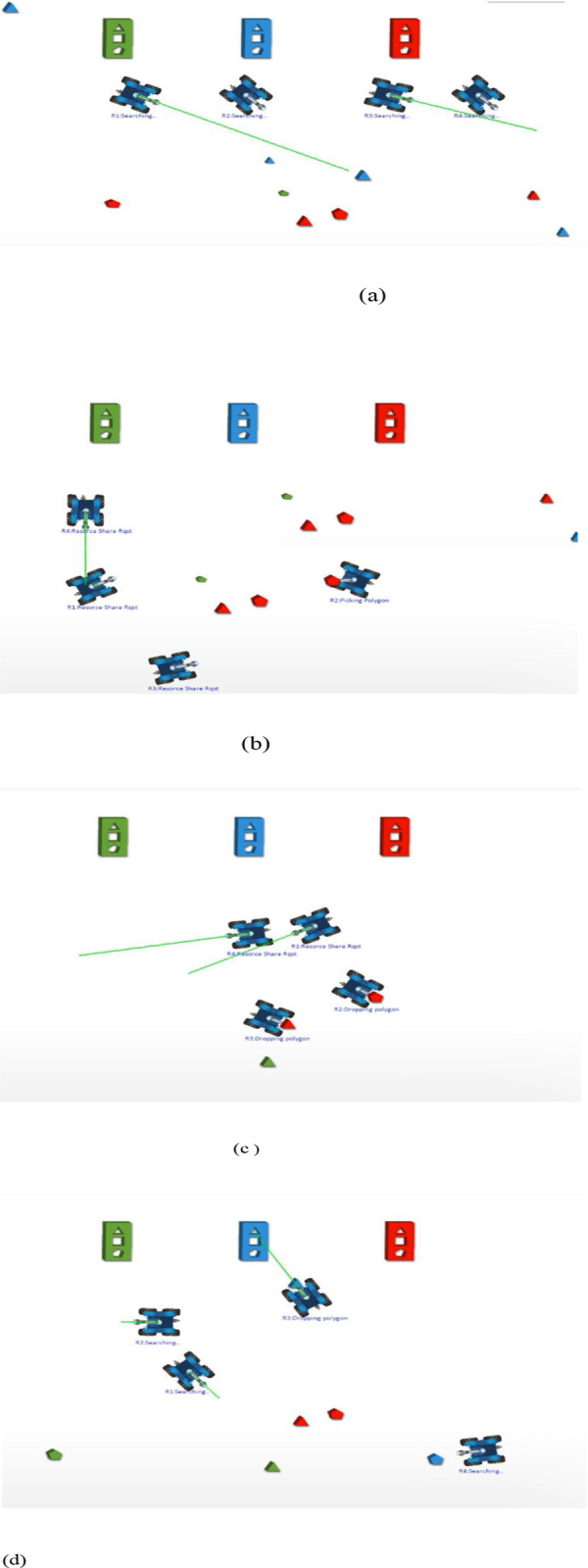
Simulation Scenario (a) Initial Position (b) *R*_4_ encounters a robotic arm failure and *R*_3_ encounters an RGB failure (c) *R*_3_ is selected by *R*_4_ for its lowest bid for robotic arm sharing (d) *R*_3_ completes the task which was detected by *R*_4_.

We validated our proposed method by testing its efficiency using a Pygame simulator with 4 robots in the environment with Python API added to interact with the environment, as shown in Fig 7. All the robots were capable of communicating and interacting with each other, and each robot had the capabilities to become the bidder or the auctioneer. The Red, Green, and Blue rectangular shapes represent the goal locations.

The experimental scenario is clearly shown in Fig 7 and 7a shows the initial position of the robots.After some time *R*_4_ encounters a \ronotic arm failure where as *R*_3_ encounters an RGB failure. *R*_4_ initiates an auction because it defficiency level is less than the acceptable defficiency level. All the robots send their bids.The lowest bid is sent by *R*_3_ hence the partially completed task is allocated to *R*_3_.

### 4.2 Real experimentation

**Fig 8:**
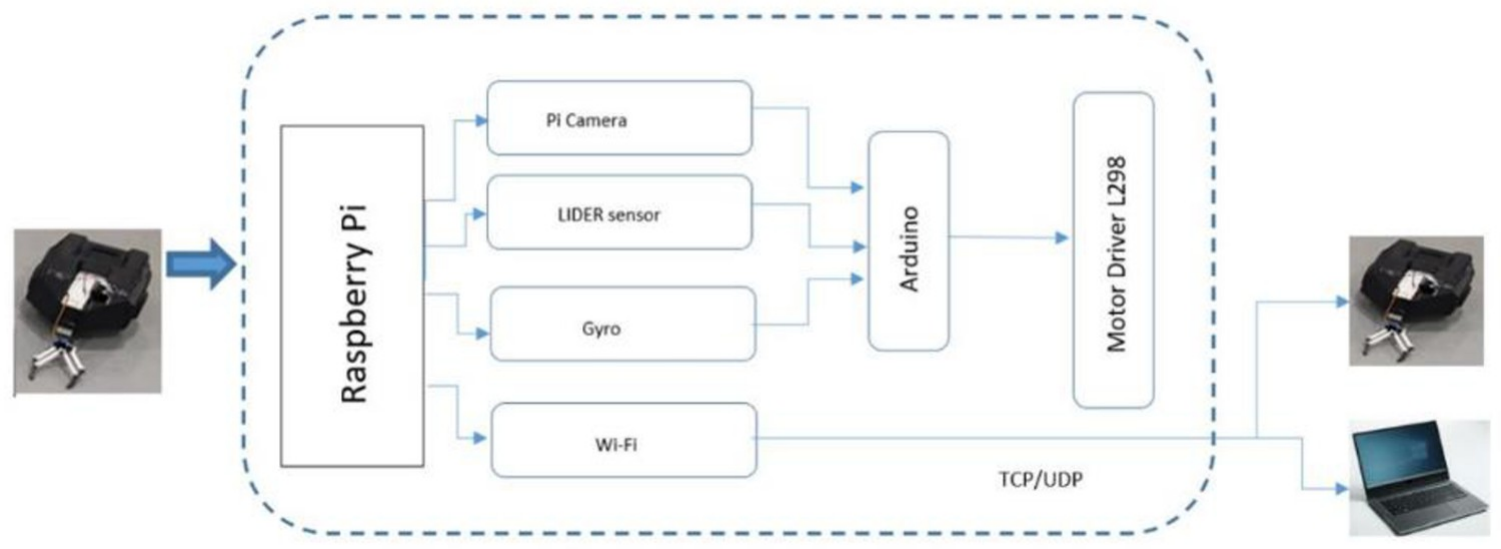
Internal hardware and resources of a robot.

**Fig 9:**
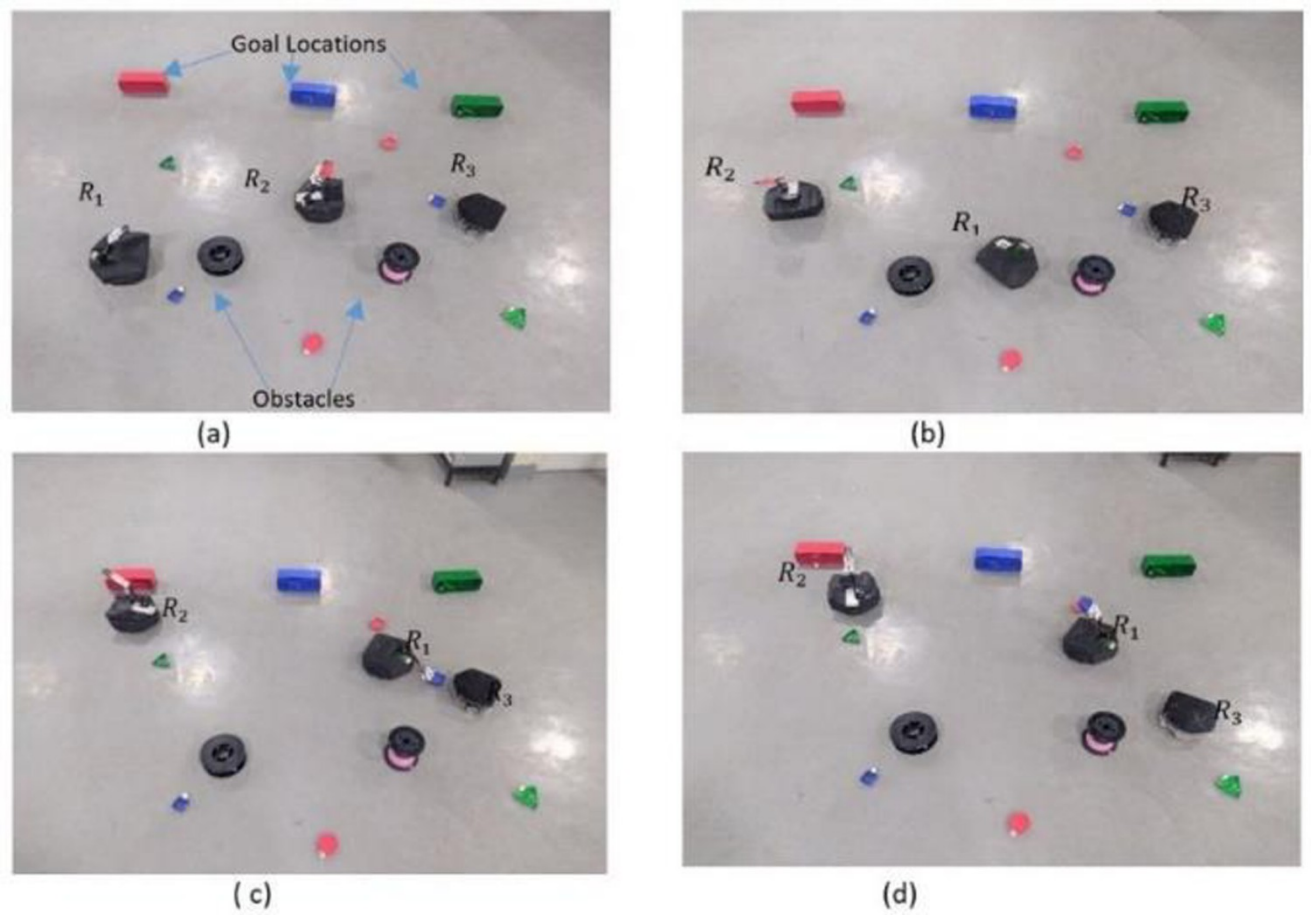
Real Experimentation (a) Robotic arm absence in *R*_3_ (b) Resource sharing request received by *R*_1_ *and R*_2_ (c)Lowest Bid of *R*_1_ accepted by *R*_3_ (d) Task completion by *R*_1_.

The onboard processor, Raspberry Pi, fully executed the implementation framework to reveal the task allocation mechanism as shown in Fig 8. Each robot was capable of communicating with other robots in the system through a WiFi module installed on the Raspberry Pi using the communication protocol TCP/UDP. The internal structure of the real robot along with the virtual viewer is shown in Fig 9 for clear description of the internal resources of the robot. Using serial communication, an Arduino connected to the Raspberry Pi controlled the Motor Driver L298.

To perform the real experimentation, a server that served as a centralized monitor was created on quad processor Intel i7 7700HQ, 16 GB RAM, and NVIDIA GeForce GTX 1080 GPU using the Socket library Python, with all the robots connected to the server using Multithreading. The Graphical User Interface (GUI) was visualized using the VNC viewer to perceive the status of each robot and tasks in the system to generate a global view of the system, as shown in Fig 8.

In the experiment, shown in Fig 9, the robot *R*_3_ was considered to have a Physical resource absence. More specifically, the robotic arm/Gripper was observed whereas *R*_1_ encountered an RGB failure. The robot *R*_3_ detected the task with RGB and recognized it using the camera and image recognition capability and planned to take it to the goal location but did not have the gripper to transport it to the goal location. A request was generated by *R*_3_ which was received by *R*_2_ *and R*_1_. Both the robots sent their bids for sharing their capabilities for the required task whereas the lowest bid was sent by *R*_1_, which was declared as the winner.

### Validation and benchmarking

The results are validated with benchmark Lee et al. [38], the task allocation with Deep Q-Learning incorporation Dai et al. [39], and Decentralized Task Allocation Ryck et al. [40]. We aim to minimize the total cost of executing tasks to complete more tasks with limited resources and task completion despite faults in robot’s capabilities, also decreasing the system’s communication burden. To achieve both purposes, the tunable parameter α was set at 0.5.

The cost is calculated in seconds considering the distance between the two points and the robot’s velocity (s/v) and the time it needs to release its occupied resources. Random faults were generated in the robot’s capabilities which changed the net operational robots in the system, as in Eq (9). Also the Instantaneous Task-Robot ratio as given in Eq (10) is utilized to validate the proposed mechanism.

## 5.Results and discussion

### 5.1 Time consumed in completing all the tasks

**Fig 10:**
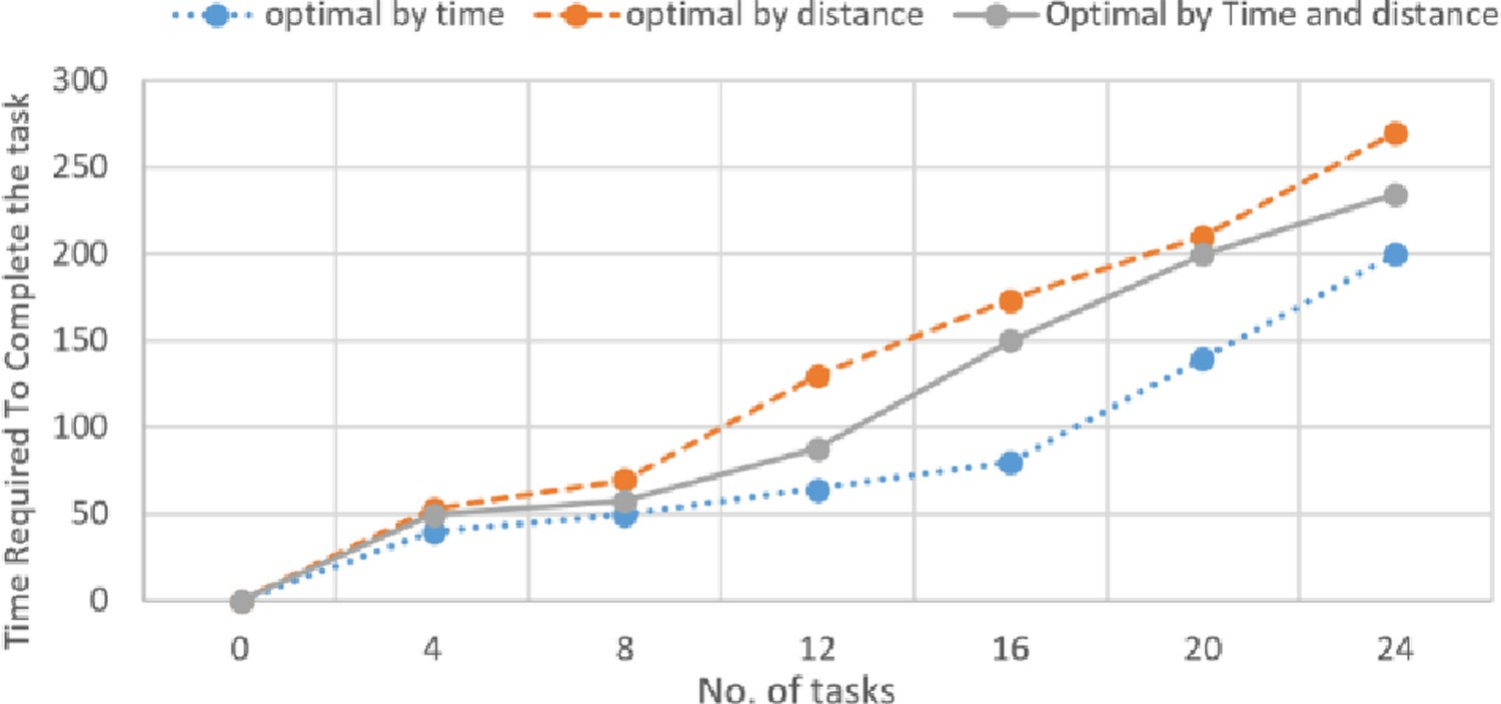
Time required for task completion with different objectives.

The time required to complete the total tasks with different objectives is calculated as shown in Fig 10.

**Fig 11:**
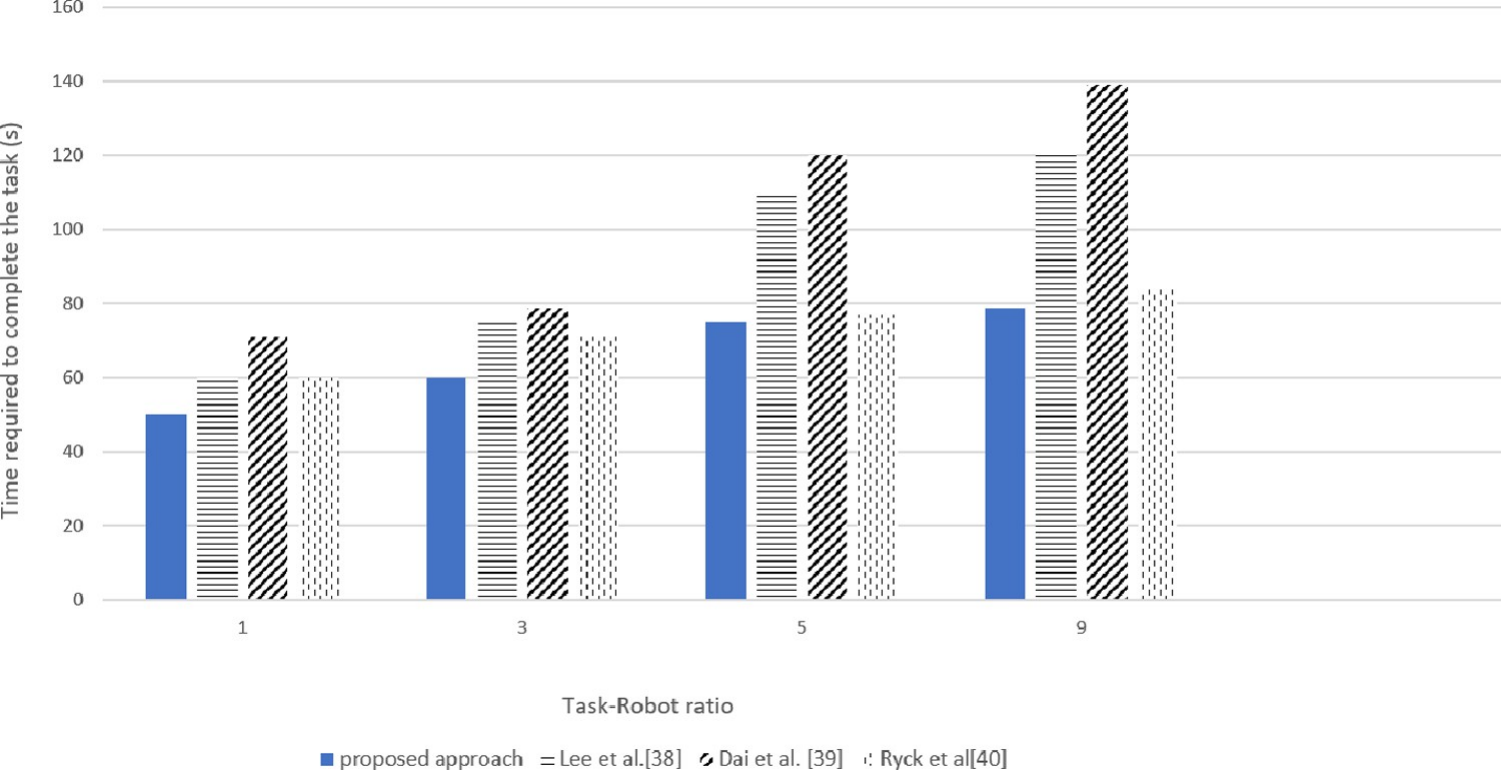
Simulated results for total completion time for various tasks-robot ratios.

**Fig 12:**
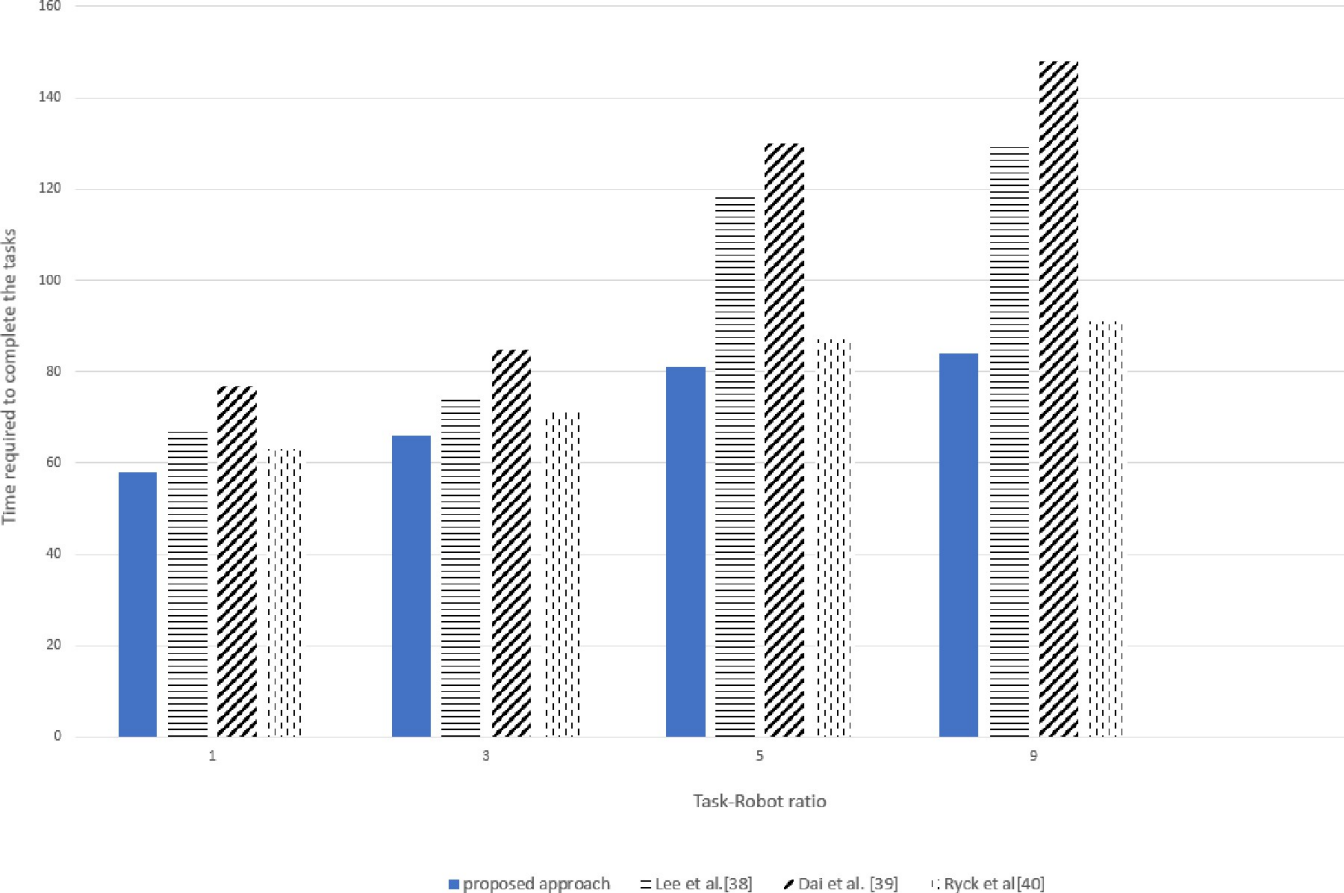
Time required for various task-robot ratio with real experimentation.

As per the user constraint or the task deadline requirement, the value of α is updated. When the deadline is the main concern, the MiniMax objective is employed by incorporating the value of α = 1 or closer to 1. Hence, the robots that can share their resources with minimum time send their bids, reducing unnecessary computations in the system. When the time deadline is not a concern the value of α = 0.5 is employed which increases the overall number of task completion and the distance travelled by the robots. However, when the Task-Robot ratio was increased to 9, an exponential increase in time consumption was observed as shown in Figs 11 and 12.

The time required by the benchmark techniques of task allocation Ryck at al [40], Dai et al. [39], Lee et al [38] with the same objective value achievement is shown in Fig 11.

The proposed approach improved the overall time completion time while considering both objectives minimization. Although the task performed by the system is simply provided all the sensor measurements and the actuators are working ideally and there are no external disturbances. However, a small difference in the simulated and real experimentation was observed in target position estimations due to slight difference in movement of robot and transportation. The difference in simulated and real experimentation values for various task-robot ratios was 4.5–5% which can be observed in Figs 11 and 12. An exponential increase in total time was observed when the task-robot ratio was increased to 9 due to vigorous sharing of resources.

### 5.2 Resource utilization

**Fig 13:**
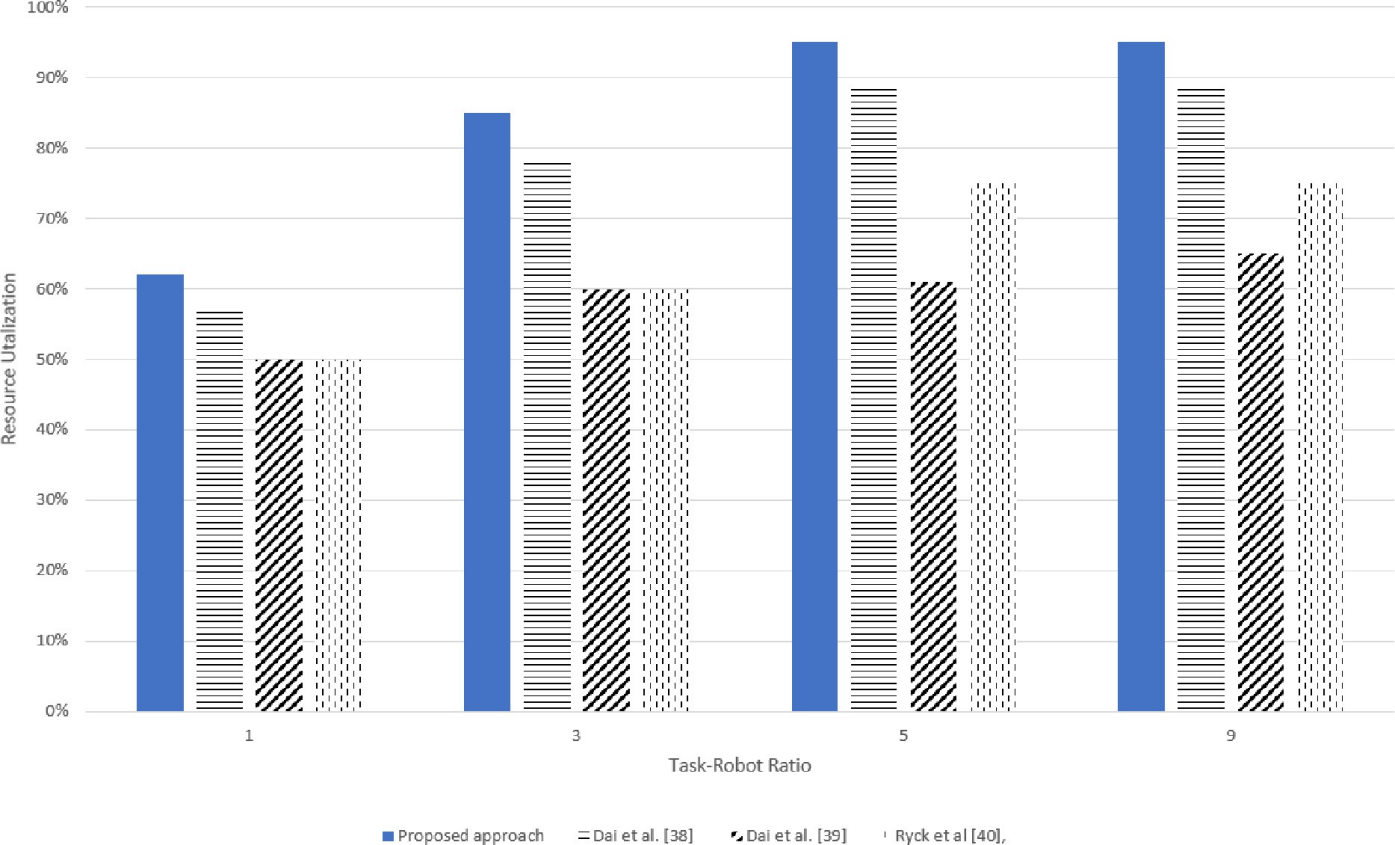
Simulation results for resource utilization for various task-robot ratio.

**Fig 14:**
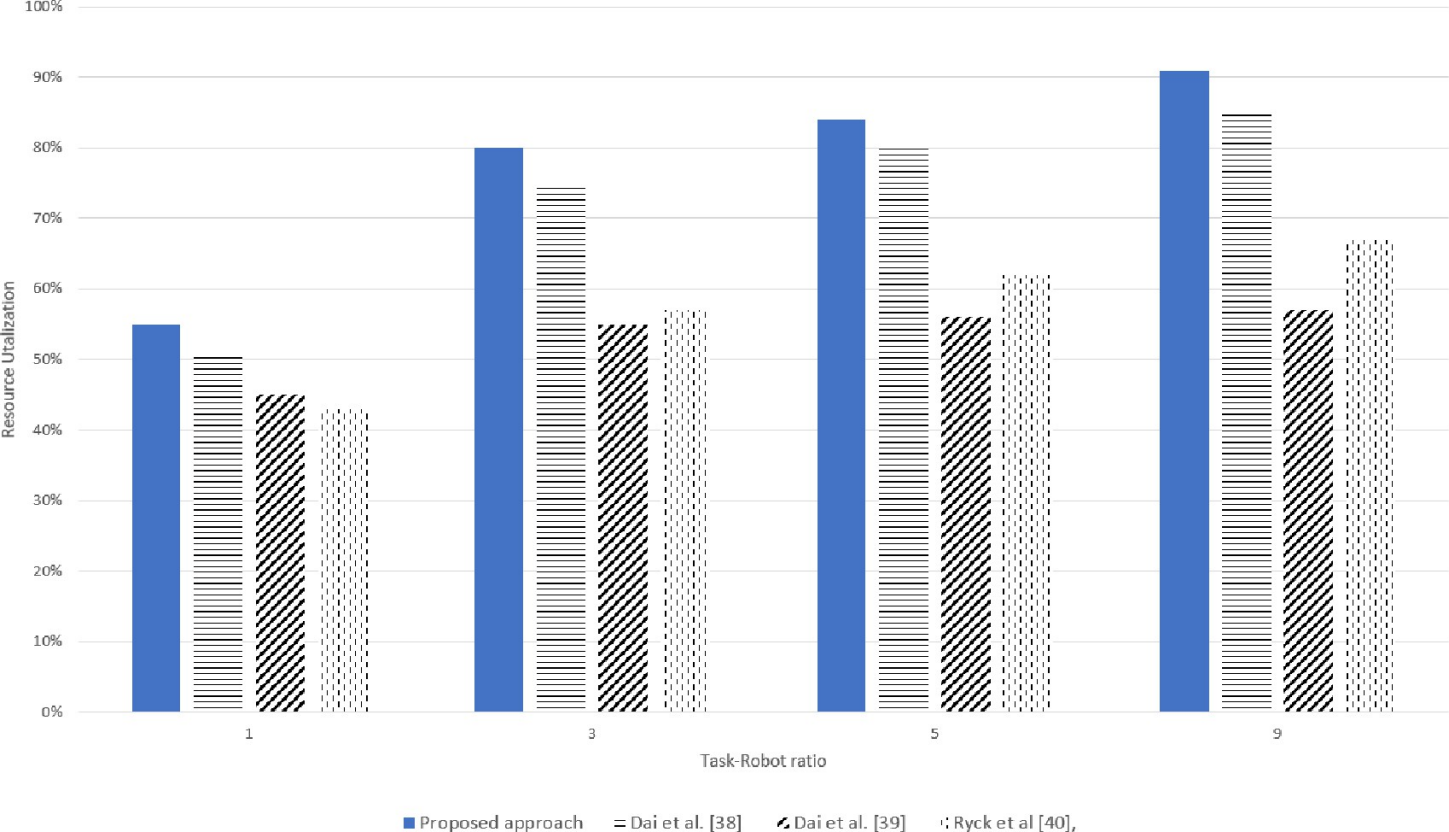
Real experimentation for resource utilization for various task-robot ratios.

Efficient resource utilization is an important parameter in evaluating a methodology. In the proposed mechanism, each experiment introduced an overall 5% resource failures or absence of the required resources to analyze resource utilization. Around 15% higher resource utilization was observed when only the soft resources failures occurred in the system and there was no hard resource failure in the system. However, enhanced resource utilization was perceived in both types of resource failures compared to the already developed techniques, as shown in Figs 13 and 14.

In the Resource-Based Task allocation Dai et al. [38], the robot’s resources were considered for task allocation however, the robots consumed most of the time in resource management, and the resource filament was rendezvous, whereas in the proposed mechanism, the deficient resources could be attained from an optimal location which increased the resource utilization and also the mission did not need to be reorganized.

Also, in Dai et al. [38] when a robot was partially damaged during task execution, the task was declared undone and needed to be reallocated to another robot the same applied to Ryck et al [40], Dai et al. [39]. Contrary to this in the proposed approach, when the robot was partially damaged, it could request for only the failed resource without re-announcing the entire task, provided the deficiency was not less than the *AIV*(*i*). The highest resource utilization of 96% was observed when the Task-Robot raised to 9 in simulation experimentation however during real experimentation there was a slight difference and the utilization reduced to 92%.

### 5.3 Communication overhead

Compared to other techniques, the proposed approach reduced the communication burden on the system, as the robots generated the requests only in case of resource absence and when the deficiency was under the roof of *AIV*(*i*).

**Fig 15:**
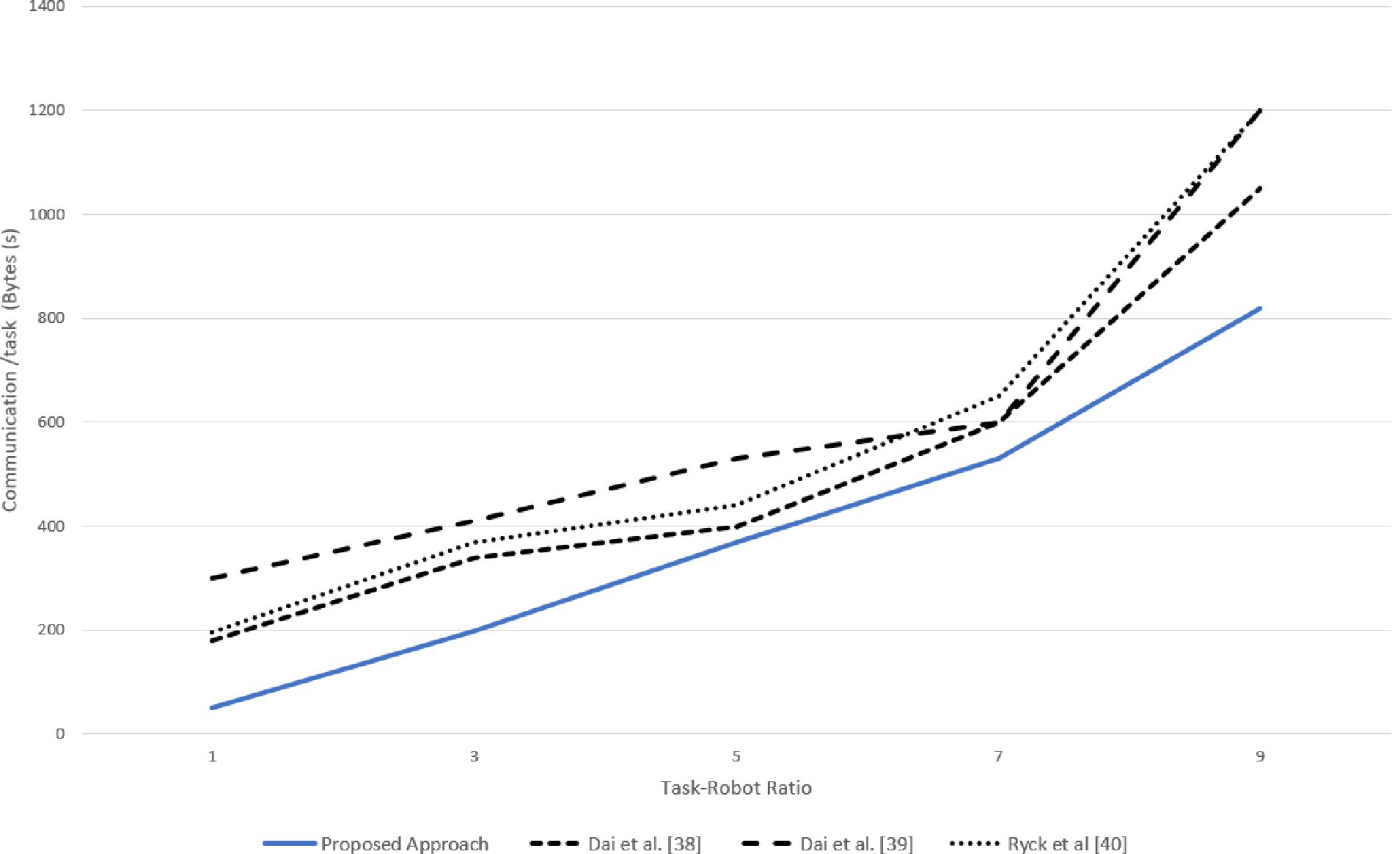
Simulation results for communication burden for various task-robot ratio.

**Fig 16:**
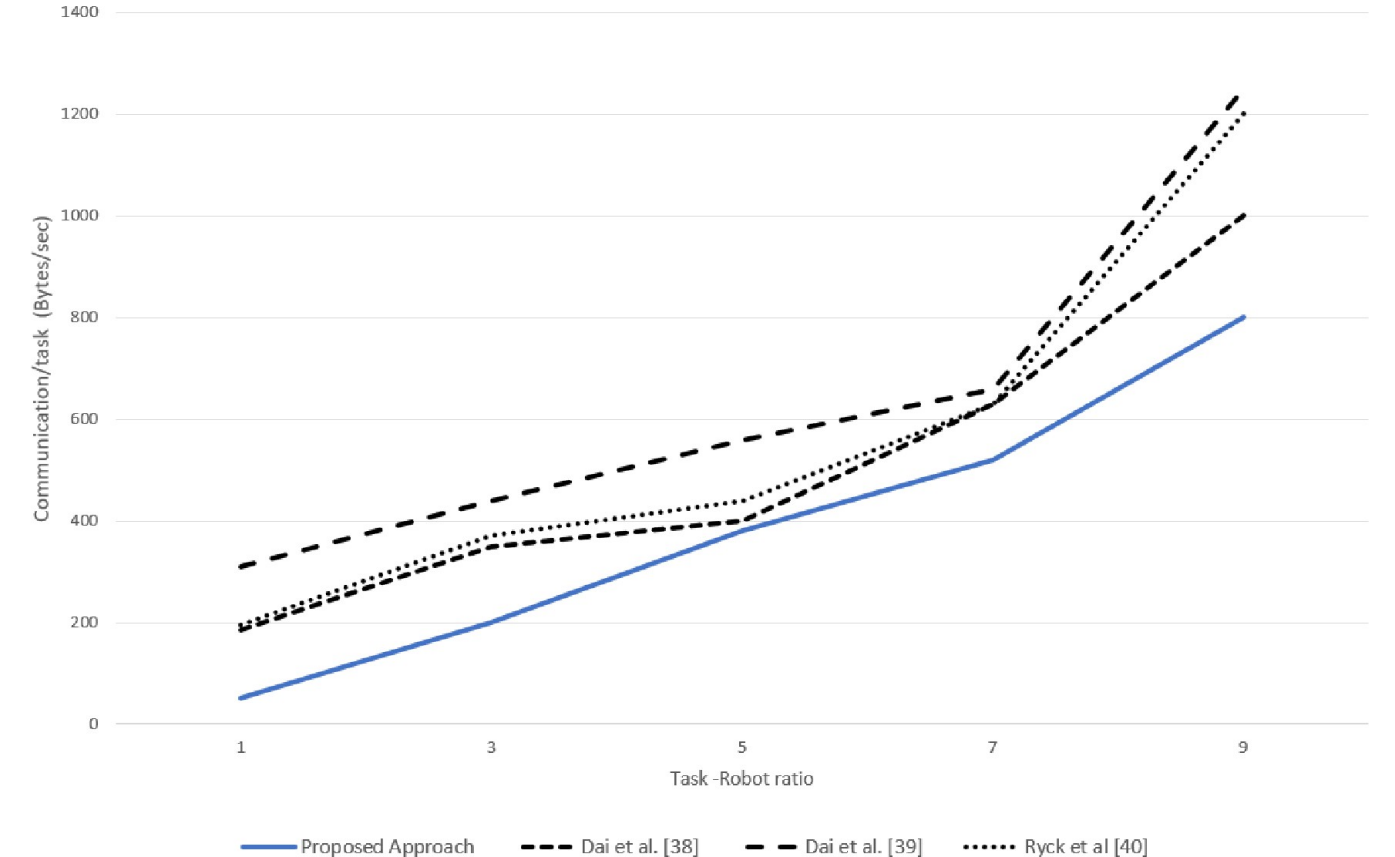
Real experimentation results for average communication overhead per task for various task-robot ratio.

A very slight difference (1–2%) in the simulated and real experimentation was observed in terms of communication overhead for each task -robot ratio in the system, as can be seen in Figs 15 and 16.The robot could not proceed further in case of resource failure Ryck at al [40], and the task needed to be re-announced and reallocated, whereas, in the proposed approach, a request was made only for the failed resources and only the robots having the required resource responded which reduced the communication burden on the entire system. Furthermore, in the task allocation proposed in Dai et al.[38] reduced communication burden; however it still had 16.7% more communication burden on the system as compared to the proposed approach, as shown in Fig 16.

## Conclusion and future work

This paper presented an efficient Task Allocation algorithm that allowed the robots to adjust their behavior due to some fault occurrence, environmental changes, or other robot’s actions to increase the overall system performance. The resource sharing technique was incorporated in task allocation algorithm to compensate the resource shortage in certain robots. The robots accepted the tasks despite resource shortage provided the resource shortage was below the threshold level. Adaptive Incentive value, accumulated the information of entire system’s resources and the resource shortage notifications into a single numerical value which served as a criterion for task acceptance. Auctions were initiated with objective minimization, in case of resource shortage relevant to the accepted task, which reduced the overall completion time in case of resource shortage. The resource sharing using the dynamic global threshold approach and the objective minimization not only reduced the task execution time but also the search time for task. The resource utilization was increased due to sharing of the temporarily free resources of the robots along with this the centralized unit was aware of the available resources of the system The proposed approach was validated through simulations using the Pygame software. To further authenticate the proposed method real robot experimentation were rigorously performed.

The proposed approach can be very effective in many areas such as firefighting, surveillance, and patrolling. This approach is specially very beneficial in situations like the pandemic of COIVID 19 when robots were deployed in hospitals and there was shortage of robots and their capabilities, and a minor fault in the robot capabilities could halt the entire process and the tasks could miss their deadline. Also a limited men power was available to fix the faults in robots. In such situations, the developed resource sharing can serve as the best solution to continue the tasks without disruption.

In the proposed approach, the path cost and the time required was assumed to be deterministic during the bidding phase. In the future, we will introduce the uncertainty and routing information in the bidding process because the time required to share the requested resource may be uncertain due to crowdedness or obstacles in the path. Further priorities would be introduced for different tasks to see their effect on the sharing mechanism.
